# Diagnostic accuracy of automated 3D volumetry of cardiac chambers by CT pulmonary angiography for identification of pulmonary hypertension due to left heart disease

**DOI:** 10.1007/s00330-022-08663-0

**Published:** 2022-03-10

**Authors:** Claudius Melzig, Thuy Duong Do, Benjamin Egenlauf, Sasan Partovi, Ekkehard Grünig, Hans-Ulrich Kauczor, Claus Peter Heussel, Fabian Rengier

**Affiliations:** 1 Clinic for Diagnostic and Interventional Radiology, Heidelberg University Hospital, Im Neuenheimer Feld 420, 69120 Heidelberg, Germany; 2 Translational Lung Research Center Heidelberg (TLRC), Member of the German Center for Lung Research (DZL), University of Heidelberg, Im Neuenheimer Feld 420, 69120 Heidelberg, Germany; 3 Centre for Pulmonary Hypertension, Thoraxklinik at Heidelberg University Hospital, Röntgenstraße 1, 69126 Heidelberg, Germany; 4 Department of Interventional Radiology, Cleveland Clinic Main Campus, 9500 Euclid Avenue, Cleveland, OH 44195 USA; 5 Department of Radiology, Thoraxklinik at Heidelberg University Hospital, Röntgenstraße 1, 69126 Heidelberg, Germany

**Keywords:** Pulmonary hypertension, Pulmonary artery, Heart, Computed tomography angiography

## Abstract

**Objectives:**

To assess diagnostic accuracy of automated 3D volumetry of cardiac chambers based on computed tomography pulmonary angiography (CTPA) for the differentiation of pulmonary hypertension due to left heart disease (group 2 PH) from non-group 2 PH compared to manual diameter measurements.

**Methods:**

Patients with confirmed PH undergoing right heart catheterisation and CTPA within 100 days for diagnostic workup of PH between August 2013 and February 2016 were included in this retrospective, single-centre study. Automated 3D segmentation of left atrium, left ventricle, right atrium and right ventricle (LA/LV/RA/RV) was performed by two independent and blinded radiologists using commercial software. For comparison, axial diameters were manually measured. The ability to differentiate group 2 PH from non-group 2 PH was assessed by means of logistic regression.

**Results:**

Ninety-one patients (median 67.5 years, 44 women) were included, thereof 19 patients (20.9%) classified as group 2 PH. After adjustment for age, sex and mean pulmonary arterial pressure, group 2 PH was significantly associated with larger LA volume (*p* < 0.001), larger LV volume (*p* = 0.001), lower RV/LV volume ratio (*p* = 0.04) and lower RV/LA volume ratio (*p* = 0.003). LA volume demonstrated the highest discriminatory ability to identify group 2 PH (AUC, 0.908; 95% confidence interval, 0.835–0.981) and was significantly superior to LA diameter (*p* = 0.009). Intraobserver and interobserver agreements were excellent for all volume measurements (intraclass correlation coefficients 0.926–0.999, all *p* < 0.001).

**Conclusions:**

LA volume quantified by automated, CTPA-based 3D volumetry can differentiate group 2 PH from other PH groups with good diagnostic accuracy and yields significantly higher diagnostic accuracy than left atrial diameter.

**Key Points:**

• *Automated cardiac chamber volumetry using non-gated CT pulmonary angiography can differentiate pulmonary hypertension due to left heart disease from other causes with good diagnostic accuracy*.

• *Left atrial volume yields significantly higher diagnostic accuracy than left atrial axial diameter for identification of pulmonary hypertension due to left heart disease without time-consuming manual processing*.

**Supplementary Information:**

The online version contains supplementary material available at 10.1007/s00330-022-08663-0.

## Introduction

Pulmonary hypertension (PH) is a hemodynamic condition defined as a pathological increase of the mean pulmonary arterial pressure (mPAP) at rest measured by right heart catheterisation (RHC) [1]. The World Health Organization (WHO) classification of PH differentiates five groups based on similarities in aetiology, hemodynamic profile, clinical findings and treatment strategy [2]. Hemodynamically, PH can be subdivided into pre-capillary, post-capillary or combined pre- and post-capillary PH [3, 4].

PH due to left heart disease (WHO group 2 PH), a form of post-capillary PH, is a frequent complication of left heart disease (LHD) and associated with worse prognosis [5, 6]. Management of group 2 PH differs from that of other PH groups and should focus on treatment of the underlying cardiac disorder [3]. The diagnosis of group 2 PH is primarily defined by an elevated pulmonary arterial wedge pressure (PAWP) > 15 mmHg at rest measured by RHC [1]. However, guidelines recommend that estimation of clinical pre-test probability for LHD based on non-invasive parameters should precede invasive catheterisation for improved differentiation of group 2 PH from other causes and improved indication of invasive right and left heart catheterisation [3].

Computed tomography (CT), especially CT pulmonary angiography (CTPA), is frequently acquired in patients with suspected PH to rule out pulmonary embolism and parenchymal lung disease. Previous studies suggested good diagnostic accuracy of enlarged left atrial diameter and area measured on CTPA for identification of group 2 PH [7–9]. However, volumetry of cardiac chambers might be more accurate and reliable in detecting cardiac chamber enlargement [10]. Technical advances have enabled automated volumetry of cardiac chambers based on CTPA [11–13], alleviating the need for time-consuming manual processing. Only a limited number of studies investigated the ability of cardiac chamber volumes to differentiate group 2 PH from other causes [7, 14, 15] without comparison to manual diameter measurements within the same patient cohort, to our knowledge.

The purpose of this study was to assess the diagnostic accuracy of automated 3D volumetry of the cardiac chambers based on CTPA for the differentiation of WHO group 2 PH from other PH groups (non-group 2 PH). Furthermore, we aimed to test our hypothesis that automated 3D volumetry yields higher diagnostic accuracy than manual diameter measurements of cardiac chambers.

## Materials and methods

### Patients

**Fig. 1:**
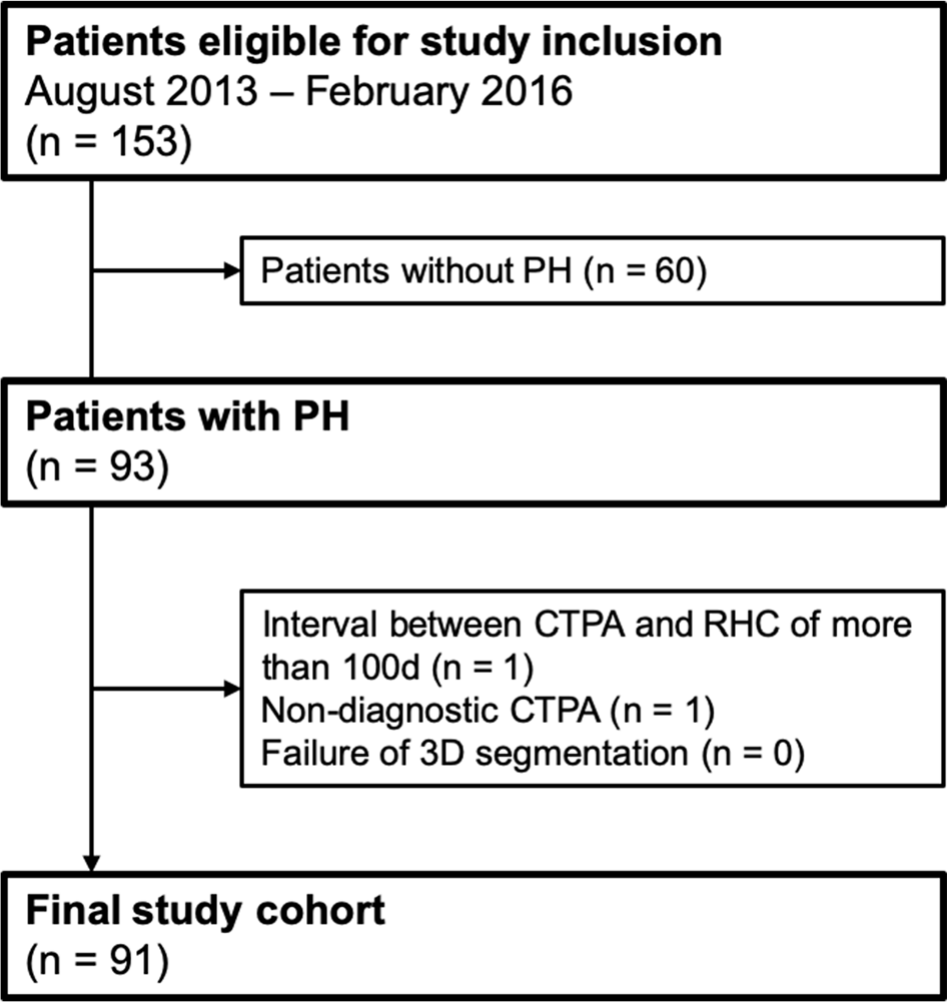
Flow chart of the study

The retrospective, single-centre study was approved by the institutional review board, and patient informed consent was waived. Records of all patients who underwent RHC and CTPA for the diagnostic workup of PH in our institution between August 2013 and February 2016 were reviewed. CTPA was performed to diagnose or rule out underlying causes, in particular chronic thromboembolic disease. Diagnosis and classification were made according to the current ESC/ERS guidelines for the diagnosis and treatment of pulmonary hypertension [1]. All patients with PH confirmed by RHC were included. Exclusion criteria were an interval between CTPA and RHC of more than 100 days, non-diagnostic CTPA and failure of 3D segmentation (Fig. 1). RHC measurements (mPAP and PAWP), WHO classification and patient characteristics (sex, height, weight, age) were extracted from the records. Body surface area (BSA) was calculated according to the formula by Du Bois and Du Bois [16].

### Right heart catheterisation

RHC was performed according to guidelines [1] by two interventional pulmonologists each with more than 7 years of experience in RHC. A 7-French pulmonary artery catheter was introduced via an 8-French introducer sheath in the right internal jugular vein. Pulmonary arterial pressure measurements were recorded after zero levelling according to the guidelines [1].

### CTPA acquisition

Non-gated CTPA data were acquired using a 64-detector CT scanner (Somatom Definition AS 64, Siemens Healthineers) in inspiratory breath-hold in supine position. Protocol settings were as follows: automated tube voltage selection (80–140 kVp), automated tube current modulation (reference tube current of 100 mAs at 120 kVp), bolus tracking in the main pulmonary artery, 50 ml of iodinated contrast agent (Ultravist 300, Bayer HealthCare) followed by a saline bolus of 50 ml with identical injection rate of 3–5 ml/s depending on venous access, collimation of 64 × 0.6 mm, iterative reconstruction kernel I40f/3 and reconstructed slice thickness of 1 mm with 0.7-mm increments. Scan length and field of view were adjusted to include the whole chest of each patient.

### Image analysis

Automated segmentation and volumetry of cardiac chambers was performed using a commercially available, model-based algorithm previously validated on gated CT scans [13, 17] (CT Pulmonary Artery Analysis, Intellispace Portal V11, Philips Healthcare). The algorithm automatically computes 3D segmentations of left and right atria (LA, RA) and ventricles (LV, RV). Pulmonary veins and the left atrial appendage were automatically excluded from the LA segmentation. Two board-certified radiologists with 6 and 8 years of experience in cardiovascular imaging reviewed segmentations in an individual random order and blinded to any clinical data, and manually corrected visible deviations from the chambers’ contours using the brush tools provided by the same software. The average of the measurements by the two radiologists was used for further analysis. To assess intraobserver agreement, one of the two radiologists repeated the image analysis after 12 months in half of the patients.

Diameter measurements of all cardiac chambers on axial slices of the CTPA data were performed by one of the board-certified radiologists in a different random order, again blinded to any other measurements and clinical data. LV and RV diameters were measured perpendicular to the interventricular septum on the slice on which the respective ventricle appeared largest [18]. LA diameter was determined as the largest anterior-posterior-diameter on the slice in the middle 50% of the craniocaudal extension of the left atrium [19]. RA diameter was measured as the largest diameter parallel to the tricuspid valve plane, excluding right atrial appendage and coronary sinus [20]. Additionally, contrast attenuation in Hounsfield units (HU) of the LA was assessed by placing a 300 mm^2^ circular region of interest in the centre of the LA on the same slice as the diameter measurement.

### Statistical analysis

Normal distribution was tested using the Shapiro-Wilk test and QQ-plots. Normally distributed continuous variables are described by mean ± standard deviation and 95% confidence intervals (CI), non-normally distributed continuous variables by median and interquartile range (IQR). Categorical variables are given as numbers and percentages. Interobserver and intraobserver agreements were assessed by calculation of intraclass correlation coefficients using a two-way mixed model testing for absolute agreement. Correlation between LA contrast attenuation and the segmentation error was analysed using Pearson’s correlation.

Comparisons between group 2 PH and non-group 2 PH patients were performed using the chi-square test for categorical variables, the two-sided *t*-test for normally distributed continuous variables and the Mann-Whitney *U* test for non-normally distributed continuous variables. ROC analyses were performed to assess the ability to differentiate group 2 PH from non-group 2 PH, and AUCs were calculated. Statistical differences of AUCs between volumetric and axial measurements were assessed using the two-sided test by DeLong et al for paired data [21]. All analyses were also performed for BSA-corrected volume and diameter measurements.

Linear correlation with the logit of each cardiac chamber measurement was confirmed and binary multiple logistic regression analysis was conducted for each cardiac chamber measurement separately, including age, sex and mPAP as potentially confounding covariates. After exclusion of significant collinearity between covariates, a binary logistic regression analysis including age and cardiac chamber volumes was performed using stepwise inclusion according to the likelihood ratio. AUC of the resulting model for the identification of group 2 PH was calculated. Sensitivities, specificities and positive and negative predictive values based on the prevalence of group 2 PH in our study cohort were calculated for the threshold values yielding the maximum Youden index for each measurement.

Using the propensity score method, pairwise matching of patients with group 2 PH to non-group 2 PH patients based on age, sex and mPAP using the nearest neighbour method was performed and group analysis repeated as described above.

A *p* value < 0.05 was considered statistically significant. All statistical analyses were accomplished using SPSS version 27.0 (SPSS Inc.) and R version 4.0.2 (R Foundation for Statistical Computing) including packages MatchIt version 4.1.0 [22] and pROC version 1.16.2 [23].

Sample size-estimation was based on Power Analysis and Sample Size (PASS 11, NCSS, LLC) [24] using the following prerequisites and assumptions: alpha level 0.05, power 0.8, prevalence of group 2 PH among the study population 20%, null hypothesis specificity 70%, alternative hypothesis specificity 90%. The calculated sample size was 39 patients including a minimum of 8 patients with group 2 PH.

## Results

### Study cohort

The patient characteristics of the final study population of 91 patients are summarised in Table 1. Nineteen patients (20.9%) were classified as group 2 PH, of which 18 (94.7%) were diagnosed with heart failure with preserved ejection fraction and 1 (5.3%) with heart failure with reduced ejection fraction. Seventy-two patients (79.1%) were identified having non-group 2 PH, of which 37 patients (51.4%) were classified as group 1 PH, 15 patients (20.8%) as group 3 PH and 20 patients (27.8%) as group 4 PH.

**Table 1:** Clinical characteristics and cardiac chamber volumes, diameters and ratios for pulmonary hypertension patients

	Group 2 PH	Non-group 2 PH	*p* value
Number of patients	19	72	
Female	12 (63.2%)	44 (61.1%)	0.87
Age (years)	76 (70–79)	67.5 (60–76)	0.004
BSA (m^2^)	1.92 ± 0.20	1.80 ± 0.21	0.03
mPAP (mmHg)	30 (27–40)	41 (30–50)	0.01
PAWP (mmHg)	19 (15–26)	11 (7–14)	< 0.001
Days between CTPA and RHC	1 (0–1)	1 (1–5)	0.07
LA volume (ml)	132.2 (112–157)	71 (61–89)	< 0.001
LV volume (ml)	97.3 (84–129)	72.1 (58–87)	< 0.001
RA volume (ml)	148.5 (106–231)	111.5 (91–175)	0.03
RV volume (ml)	163.4 (111–309)	169.4 (129–231)	0.86
RV/LV volume ratio	1.68 (1.4–2.1)	2.55 (1.6–3.4)	0.004
RA/LA volume ratio	1.21 (1.0–1.8)	1.59 (1.2–2.2)	0.005
RV/LA volume ratio	1.03 (0.9–2.2)	2.24 (1.7–3.5)	< 0.001
LA axial diameter (mm)	48 (43–59)	39 (35–44)	< 0.001
LV axial diameter (mm)	47 (38–51)	38 (33–43)	< 0.001
RA axial diameter (mm)	70 (61–79)	61 (52–71)	0.04
RV axial diameter (mm)	50 (43–65)	53 (47–60)	0.71
RV/LV diameter ratio	1.14 (0.9–1.5)	1.38 (1.2–1.7)	0.01
RA/LA diameter ratio	1.37 (1.2–1.5)	1.55 (1.4–1.9)	0.02
RV/LA diameter ratio	1.00 (0.9–1.1)	1.35 (1.2–1.6)	< 0.001

Values are presented as mean ± standard deviation for BSA, *n* (%) for sex or median (interquartile range) for all other parameters, as appropriate according to Shapiro-Wilk test of distribution. *BSA*, body surface area; *CTPA*, pulmonary computed tomography angiography; *LA*, left atrium; *LV*, left ventricle; *mPAP*, mean pulmonary arterial pressure; *PAWP*, pulmonary arterial wedge pressure; *PH*, pulmonary hypertension; *RA*, right atrium; *RHC*, right heart catheterisation; *RV*, right ventricle

### Cardiac chamber volumes and diameters

**Fig. 2:**
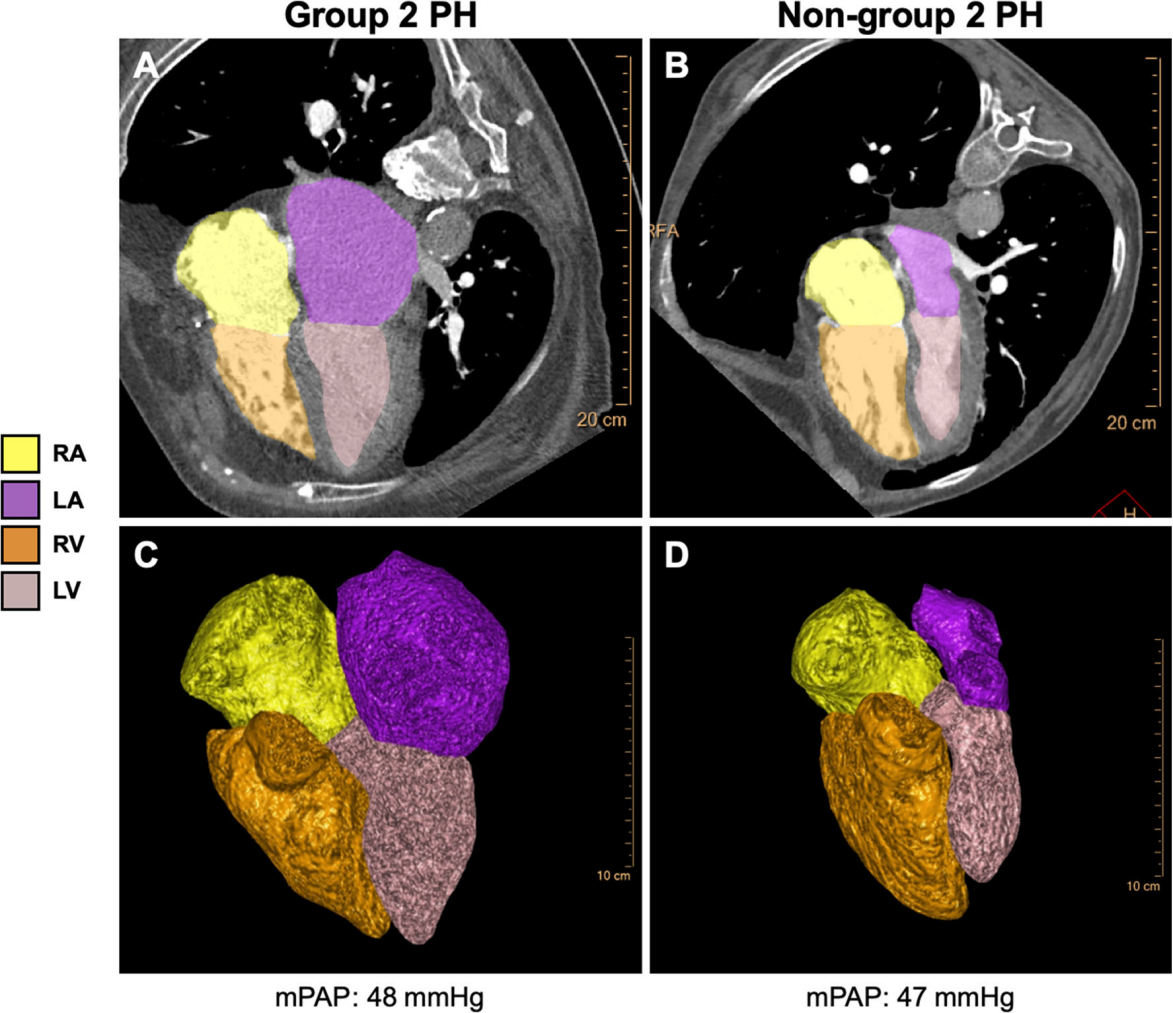
Upper row (**A**, **B**) shows four-chamber view, lower row (**C**, **D**) 3D visualisations of representative cardiac chamber segmentations in a 79-year-old female patient with group 2 pulmonary hypertension (**A**, **C**) compared to a 63-year-old female patient with group 3 pulmonary hypertension (**B**, **D**). A particular enlargement of the LA is visible in the patient with group 2 pulmonary hypertension. LA, left atrium; LV, left ventricle; mPAP, mean pulmonary arterial pressure; PH, pulmonary hypertension; RA, right atrium; RV, right ventricle

**Fig. 3:**
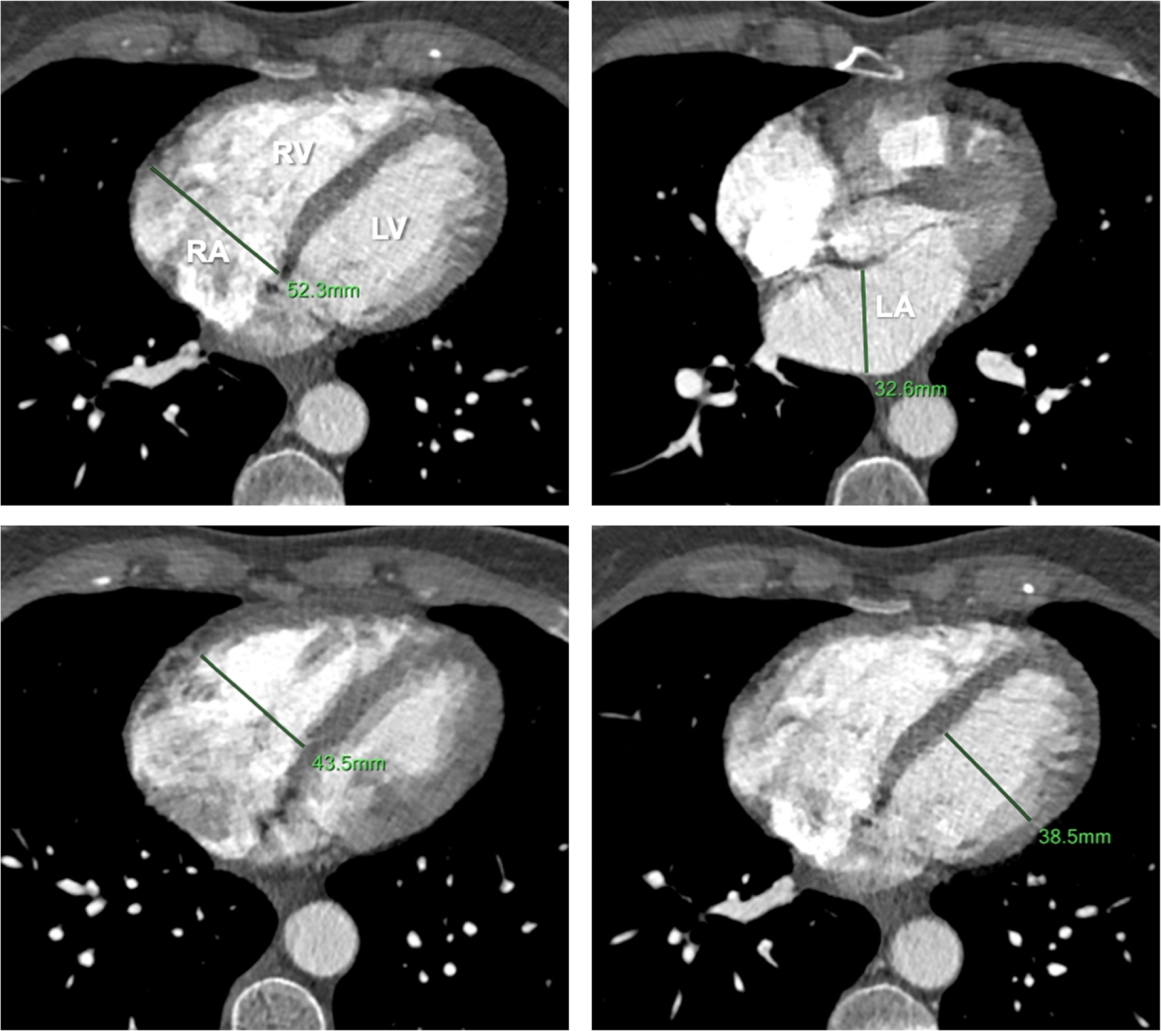
Example of manual diameter measurements of cardiac chambers on standard axial CTPA slices. LA, left atrium; LV, left ventricle; RA, right atrium; RV, right ventricle

**Fig. 4:**
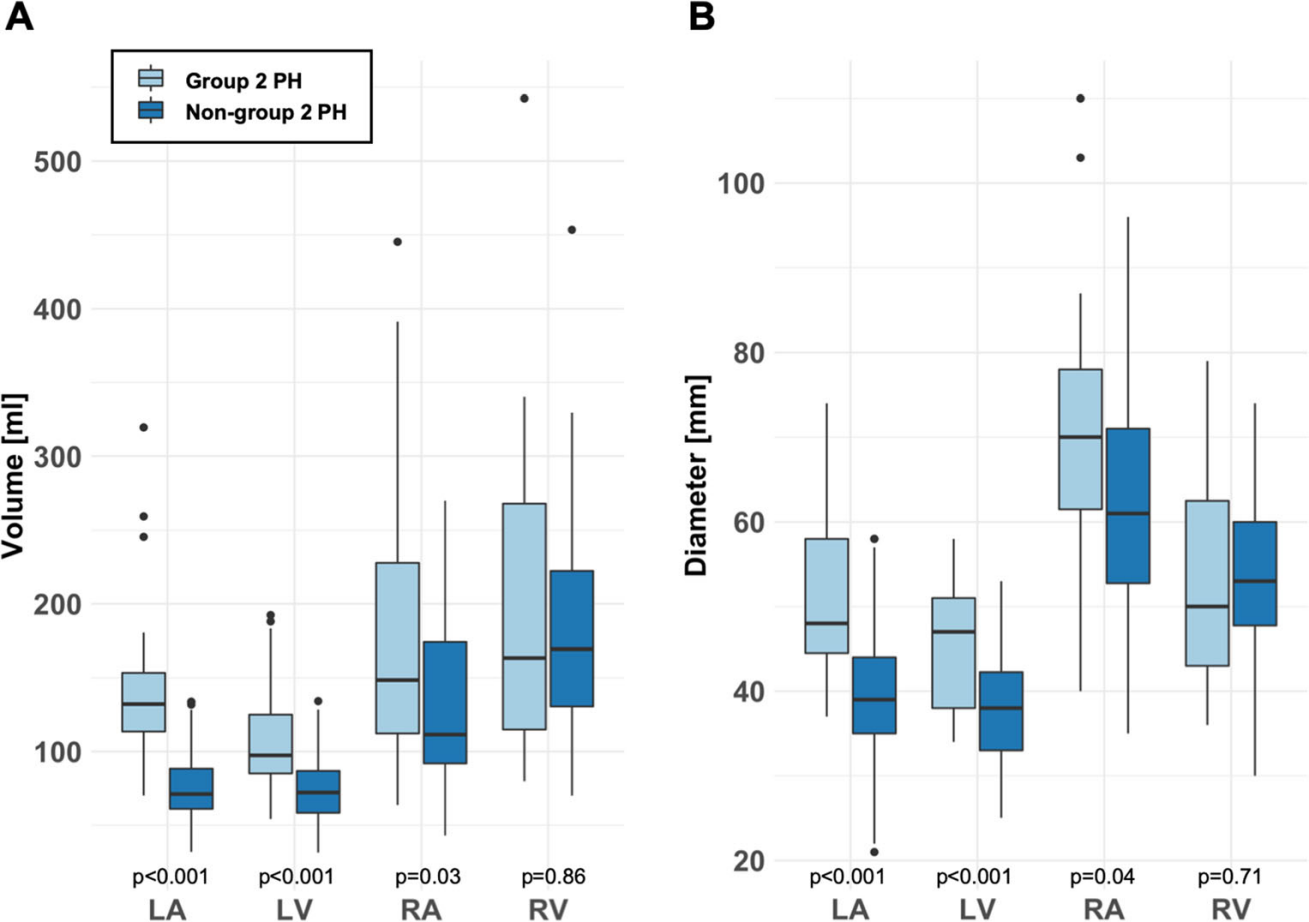
Boxplot diagrams of volumes (**A**) and axial diameters (**B**) of all cardiac chambers for group 2 pulmonary hypertension patients and non-group 2 pulmonary hypertension patients. LA, left atrium; LV, left ventricle; RA, right atrium; RV, right ventricle

Volumes and diameters of LA, LV and RA were significantly larger in patients with group 2 PH compared to non-group 2 PH, while RV/LV, RA/LA and RV/LA volume and diameter ratios were significantly lower (Table 1, Figs. 2, 3 and 4).

Interobserver and intraobserver agreements of cardiac chamber volumetry were excellent for all cardiac chambers, with intraclass correlation coefficients of 0.951/0.926 for LV volume (95% CI 0.923–0.968/0.872–0.958, both *p* < 0.001), 0.978/0.980 for RV volume (95% CI 0.967–0.986/0.954–0.990, both *p* < 0.001), 0.969/0.983 for LA volume (95% CI 0.954–0.980/0.970–0.990, both *p* < 0.001) and 0.999/0.998 for RA volume (95% CI 0.998–0.999/0.997–0.999, both *p* < 0.001). Manual adjustments of automatic segmentations were performed in 38 of 91 patients (41.8%)/96 of 364 cardiac chambers (26.4%) by reader 1 and 45 patients (49.5%)/102 cardiac chambers (28.0%) by reader 2, yielding fully automatic segmentations of 72.8% of cardiac chambers without the need of manual adjustment. In cases of manual adjustment, average time for adjusting the segmentation of one cardiac chamber was 86.7 ± 34.2 s. The segmentation error, i.e. the difference between corrected and fully automatic volumes, was small (95% CI for LA (−3.4)–(+0.4)ml/(−6.9)–(+0.4%), for LV (−3.6)–(+2.0)ml/(−5.9)–(+1.2%), for RA (−2.7)–( −0.2)ml/(−1.3)–( −0.2%) and for RV (+7.0)–(+17.9)ml/(+2.5)–(+6.0%)).

Mean LA contrast attenuation was 288.0 HU (95% CI 267.8–308.2 HU). There was only a very weak positive, statistically non-significant correlation between LA contrast attenuation and the segmentation error for LA volume (*r*^2^ = 0.021, *p* = 0.17).

### Identification of WHO group 2 pulmonary hypertension

ROC analysis showed excellent ability of LA volume to differentiate group 2 PH from non-group 2 PH with an AUC of 0.908 (95% CI 0.835–0.981) and good ability of LV volume, as well as RV/LV, RA/LA and RV/LA volume ratios (Table 2, Supplementary Table A). Cardiac chamber volumes demonstrated higher AUC values compared to axial diameters, with LA volume showing statistically significant superiority compared to LA diameter (*p* = 0.009, Table 2). Multiple logistic regression analysis demonstrated that the significance of enlarged LA volume (*p* < 0.001), enlarged LV volume (*p* = 0.001), lower RV/LV volume ratio (*p* = 0.04) and lower RV/LA volume ratio (*p* = 0.003) as predictors for group 2 PH persisted after adjustment for age, sex and mPAP (Table 3). The same was true for the respective axial diameter values (Table 3).

**Table 2:** ROC analysis of cardiac chamber volumes, diameters and ratios to differentiate group 2 pulmonary hypertension patients from non-group 2 pulmonary hypertension patients

	Volume	Axial diameter	*p* value
Left atrium (LA)	0.908 (0.835–0.981)	0.830 (0.738–0.922)	0.009
Left ventricle (LV)	0.784 (0.664–0.903)	0.745 (0.618–0.871)	0.52
Right atrium (RA)	0.664 (0.523–0.806)	0.657 (0.517–0.796)	0.89
Right ventricle (RV)	0.487 (0.317–0.657)	0.472 (0.304–0.640)	0.74
RV/LV ratio	0.717 (0.601–0.832)	0.686 (0.547–0.825)	0.61
RA/LA ratio	0.708 (0.579–0.838)	0.673 (0.544–0.802)	0.50
RV/LA ratio	0.822 (0.709–0.934)	0.804 (0.673–0.935)	0.73

Values are given as area under the curve (95% confidence interval)

**Table 3:** Cardiac chamber volumes, diameters and ratios as predictors for group 2 pulmonary hypertension after adjustment for age, sex and mean pulmonary arterial pressure

	Odds ratio	*p* value
LA volume (10 ml)	2.062 (1.383–3.074)	< 0.001
LV volume (10 ml)	1.721 (1.253–2.364)	0.001
RA volume (10 ml)	1.181 (1.059–1.318)	0.003
RV volume (10 ml)	1.099 (1.009–1.196)	0.03
RV/LV volume ratio	0.380 (0.152–0.953)	0.04
RA/LA volume ratio	0.504 (0.174–1.464)	0.21
RV/LA volume ratio	0.142 (0.039–0.510)	0.003
LA axial diameter (mm)	1.199 (1.087–1.322)	< 0.001
LV axial diameter (mm)	1.243 (1.106–1.396)	< 0.001
RA axial diameter (mm)	1.070 (1.015–1.128)	0.01
RV axial diameter (mm)	1.008 (0.944–1.076)	0.82
RV/LV diameter ratio	0.051 (0.006–0.448)	0.007
RA/LA diameter ratio	0.167 (0.023–1.205)	0.08
RV/LA diameter ratio	0.016 (0.001–0.230)	0.002

Values are given as odds ratio (95% confidence interval). *LA*, left atrium; *LV*, left ventricle; *RA*, right atrium; *RV*, right ventricle

The stepwise logistic regression analysis for identification of group 2 PH patients resulted in the final model with LA volume (*p* < 0.001) and RV/LV volume ratio (*p* = 0.02) as covariates:

$$ \mathrm{Logit}\left({\mathrm{probability}}_{\mathrm{Group}\ 2\ \mathrm{PH}}\right)=0.64\times \mathrm{LA}\ \mathrm{volume}-1.39\times \mathrm{RV}/\mathrm{LV}\ \mathrm{volume}\ \mathrm{ratio}-4.954 $$

ROC analysis demonstrated an AUC of 0.936 (95% CI 0.879–0.993). The corresponding sensitivity, specificity and positive and negative predictive values using a cut-off probability of > 0.40 yielding the maximum Youden index of 1.73 were 78.9%, 94.4%, 78.9% and 94.4%, respectively (Table 4). For LA volume/LA diameter alone, sensitivity, specificity and positive and negative predictive values using a cut-off value of ≥ 110 ml/≥ 41 mm were 78.9%/89.5%, 90.3%/62.5%, 68.2%/38.6% and 94.2%/95.7%, respectively (Table 4).

**Table 4:** Diagnostic accuracy for the differentiation of group 2 pulmonary hypertension from non-group 2 pulmonary hypertension

	Group 2 PH	Non-group 2 PH
Regression model (predicted probability)		
< 0.40	4 (21.1%)	68 (94.4%)
≥ 0.40	15 (78.9%)	4 (5.6%)
LA volume		
< 110 ml	4 (21.1%)	65 (90.3%)
≥ 110 ml	15 (78.9%)	7 (9.7%)
LA diameter		
< 41 mm	2 (10.5%)	45 (62.5%)
≥ 41 mm	17 (89.5%)	27 (37.5%)

*LA*, left atrium; *PH*, pulmonary hypertension

The above-mentioned analyses were also conducted after adjusting cardiac chamber volumes and diameters for BSA. This did not have a relevant influence on the major findings. For example, ROC analysis to differentiate group 2 PH from non-group 2 PH yielded AUC values of 0.893 for LA volume_BSA_ (95% CI 0.819–0.967), 0.761 for LV volume_BSA_ (95% CI 0.636–0.886), 0.636 for RA volume_BSA_ (95% CI 0.491–0.781) and 0.447 for RV volume_BSA_ (95% CI 0.280–0.613).

Finally, to account for differences in clinical characteristics between the two groups, pairwise matching of group 2 (*n* = 19) and non-group 2 PH patients (*n* = 19) was performed using the propensity score method (Table 5). Analyses after pairwise matching confirmed that volumes and diameters of LA, LV and RA were significantly larger in group 2 PH patients compared to matched non-group 2 PH patients, while RV/LA volume and diameter ratios were significantly lower (Table 5).

**Table 5:** Clinical characteristics and cardiac chamber volumes, diameters and ratios for group 2 pulmonary hypertension patients and propensity score–matched non-group 2 pulmonary hypertension patients

	Group 2 PH	Non-group 2 PH	*p* value
Number of patients	19	19	
Female	12 (63.2%)	11 (57.9%)	0.74
Age (years)	76 (70–79)	75 (70–78)	0.56
BSA (m^2^)	1.92 ± 1.98	1.76 ± 1.48	0.009
mPAP (mmHg)	30 (27–40)	30 (27–44)	0.71
PAWP (mmHg)	19 (15–26)	10 (7–13)	< 0.001
Days between CTPA and RHC	1 (0–1)	1 (1–5)	0.22
LA volume (ml)	132 (112–157)	69.4 (58–98)	< 0.001
LV volume (ml)	97.3 (84–129)	65.0 (59–91)	0.009
RA volume (ml)	148.5 (106–231)	103.9 (70–172)	0.046
RV volume (ml)	163.4 (111–309)	160.8 (113–199)	0.60
RV/LV volume ratio	1.68 (1.4–2.1)	1.92 (1.4–2.8)	0.20
RA/LA volume ratio	1.21 (1.0–1.8)	1.50 (1.1–2.0)	0.12
RV/LA volume ratio	1.03 (0.9–2.2)	2.05 (1.6–2.8)	0.004
LA axial diameter (mm)	48 (43–59)	39 (36–48)	0.002
LV axial diameter (mm)	47 (38–51)	37 (33–41)	0.009
RA axial diameter (mm)	70 (61–79)	61 (53–65)	0.05
RV axial diameter (mm)	50 (43–65)	50 (46–59)	0.84
RV/LV diameter ratio	1.14 (0.9–1.5)	1.32 (1.2–1.6)	0.11
RA/LA diameter ratio	1.37 (1.2–1.5)	1.53 (1.2–1.8)	0.35
RV/LA diameter ratio	1.00 (0.9–1.1)	1.20 (1.1–1.4)	0.004

Values are presented as mean ± standard deviation for BSA, *n* (%) for sex or median (interquartile range) for all other parameters, as appropriate according to Shapiro-Wilk test of distribution. *BSA*, body surface area; *CTPA*, pulmonary computed tomography angiography; *LA*, left atrium; *LV*, left ventricle; *mPAP*, mean pulmonary arterial pressure; *PAWP*, pulmonary arterial wedge pressure; *PH*, pulmonary hypertension; *RA*, right atrium; *RHC*, right heart catheterisation; *RV*, right ventricle

## Discussion

This study assessed the diagnostic accuracy of automated 3D volumetry of the cardiac chambers based on CTPA for the differentiation of WHO group 2 PH from other PH groups and demonstrated excellent ability of LA volume to differentiate group 2 PH from non-group 2 PH with an AUC of 0.908 and corresponding sensitivity, specificity and positive and negative predictive values at the maximum Youden index of 78.9%, 90.3%, 68.2% and 94.2%, respectively. LA volume was a significantly better predictor of group 2 PH compared to LA diameter as measured on axial CTPA slices. Diagnostic accuracy could be slightly improved by combining LA volume and RV/LV volume ratio resulting in an AUC of 0.936.

Correct diagnosis of group 2 PH can be challenging, especially in cases of heart failure with preserved ejection fraction, and the diagnostic pathway should be based on the pre-test probability of LHD [3]. As CTPA is frequently acquired in addition to echocardiography during diagnostic workup [3, 25, 26], including CTPA-based parameters into the estimation of pre-test probability of LHD appears to be a promising approach for translation into clinical practice and may improve confidence when deciding which patient may require invasive catheterisation. LA area and volume can also be estimated by transthoracic echocardiography using planar measurements and geometric assumptions [3, 25]. However, LA volume estimation by echocardiography has been shown to be less accurate compared to CT-based volume measurements [26].

One previous study applied the software for cardiac chamber volume analysis also used in our study in a similar clinical setting and patient cohort size [14]. This previous study found a similar AUC value of 0.92 using LA volume for identification of group 2 PH, but did not perform diameter measurements for comparison [14]. Our study not only provides confirmatory evidence for the diagnostic accuracy of LA volume in a different institution and patient cohort but also demonstrates its superiority compared to LA axial anteroposterior diameter in an intra-individual comparison. LA anteroposterior diameter is the most widely used LA measurement in clinical practice and PH research in both echocardiography and CT according to international recommendations and literature [7, 19, 27–30]. Previous studies reported AUC values of 0.71–0.83 for identification of group 2 PH by LA anteroposterior diameter, compared to 0.83 in our study cohort [7, 8].

LA transverse diameter represents an alternative but has only been investigated in a single study regarding identification of group 2 PH [8]. LA area measurements have also been proposed to differentiate group 2 PH from non-group 2 PH with reported AUC values of 0.73–0.85 being slightly higher compared to LA anteroposterior diameter, but required time-consuming manual delineation of the LA thus hampering its clinical use [7–9]. In our study, the software provided robust segmentations without the need of manual adjustment in 72.8% of cardiac chambers and tools for manual adjustments with little time and effort. Fully automatic segmentation without any manual adjustment resulted in only marginally lower diagnostic accuracy with an AUC of 0.90 for LA volume.

The advantage of volume measurements over one- or two-dimensional measurements has also been demonstrated for other body regions [31–33]. This may be attributable to the lower susceptibility to geometric variances in asymmetrical geometries, as has also been discussed for the left atrium [10]. Prospective studies are needed to confirm our findings.

This study is subject to some limitations. A retrospective study might be prone to confounders and selection bias. However, we aimed to control for potential confounders by including respective covariates in multiple regression analysis and for potential group differences in the baseline characteristics by performing propensity score matching. Diagnosis and classification of PH groups in this study were based on the current ESC/ERS guidelines [1]. While the 6^th^ World Symposium on Pulmonary Hypertension suggested some changes to the PH definitions, these suggestions have not yet been integrated into the ESC/ERS guidelines [4]. The findings of our study might differ to some degree should the new recommendations be adopted by the guidelines in the future. Furthermore, non-gated CTPA was used for cardiac chamber segmentation. The model-based algorithm was previously evaluated on gated and non-gated CT scans and found to be accurate and highly reliable [13, 14, 17]. Gated CT scans, however, are less frequently acquired in clinical routine, may have higher radiation exposure and be less beneficial for LA volume measurements given the smaller amount of LA motion during the cardiac cycle compared to LV or RV motion. Contrast enhancement of the cardiac chambers was sufficient for reproducible measurements as demonstrated by the excellent interobserver and intraobserver agreements, as well as the small segmentation error and its non-significant correlation with LA contrast attenuation. The 95% CI of LA attenuation (267.8–308.2 HU) was well above the threshold of 210 HU for MPA attenuation considered as a diagnostic CTPA scan [34]. Of note, we observed a slight tendency of the algorithm to underestimate RV volume in PH patients. Finally, in accordance with previous studies, the present study did not address patients with isolated post-capillary PH and patients with combined pre- and post-capillary PH separately [7–9].

In conclusion, automated 3D volumetry of cardiac chambers based on non-gated CTPA, in particular LA volume, can differentiate WHO group 2 PH patients from other PH groups with good diagnostic accuracy. LA volume yields significantly higher diagnostic accuracy than LA diameter as measured on axial CTPA slices. If proved reliable in prospective studies, cardiac chamber volumetry based on CTPA may improve non-invasive classification of PH due to LHD and thereby optimise diagnostic pathways.

## Supplementary Information

ESM 1 (DOCX 17 kb)

## Abbreviations

CTPA: CT pulmonary angiography
LA: Left atrium
LHD: Left heart disease
LV: Left ventricle
PH: Pulmonary hypertension
RA: Right atrium
RHC: Right heart catheterisation
RV: Right ventricle

## Declarations

### Guarantor

The scientific guarantor of this publication is Fabian Rengier.

### Statistics and biometry

One of the authors has significant statistical expertise.

### Study subjects or cohorts overlap

The present study forms part of an ongoing institutional investigation on imaging in patients with known or suspected PH based on an institutional database. Seventy patients (out of the 91 patients included in the present study) were included in a prior study analysing the pulmonary arteries, but not the cardiac chambers. None of the measurements reported in the present study formed part of this prior study. Reference of this prior study: Melzig, C., Wörz, S., Egenlauf, B. et al Combined automated 3D volumetry by pulmonary CT angiography and echocardiography for detection of pulmonary hypertension. *Eur Radiol* 29, 6059–6068 (2019). 10.1007/s00330-019-06188-7. The study cohort also partially overlaps with a further study based on the same institutional database, including cardiac chamber measurements of the present study, analysing a different outcome. Reference of an interim analysis of the latter study: Fink, M.A., Melzig, C., Egenlauf, B. et al Machine learning-based cardiac chamber segmentation in CTPA for the noninvasive detection of pulmonary hypertension. ECR 2020 Book of Abstracts, p. 575, Insights Imaging 11(Suppl 1), 34 (2020). 10.1186/s13244-020-00851-0.

### Methodology

• retrospective

• diagnostic or prognostic study

• performed at one institution
