# Supplementary material for: Diagnostic accuracy of automated 3D volumetry of cardiac chambers by CT pulmonary angiography for identification of pulmonary hypertension due to left heart disease
Source: Eur Radiol. 2022 Mar 10;32(8):5222–32. doi: 10.1007/s00330-022-08663-0 (PMC9279230; doi:10.1007/s00330-022-08663-0)
Supplement: Supplementary file 1 — (DOCX 17 kb) [file 330_2022_8663_MOESM1_ESM.docx]

# Supplementary Material

Supplementary Table A

ROC analysis of fully automatic cardiac chamber volumes to differentiate group 2 pulmonary hypertension patients from non-group 2 pulmonary hypertension patients in comparison with axial diameters

|  | **Fully automatic volume** | **Axial diameter** | **P-value** |
| --- | --- | --- | --- |
| Left atrium (LA) | 0.897 (0.816-0.978) | 0.830 (0.738-0.922) | 0.035 |
| Left ventricle (LV) | 0.746 (0.616-0.877) | 0.745 (0.618-0.871) | 0.98 |
| Right atrium (RA) | 0.665 (0.521-0.809) | 0.657 (0.517-0.796) | 0.87 |
| Right ventricle (RV) | 0.504 (0.331-0.678) | 0.472 (0.304-0.640) | 0.89 |
| RV/LV ratio | 0.698 (0.585-0.811) | 0.686 (0.547-0.825) | 0.85 |
| RA/LA ratio | 0.689 (0.555-0.823) | 0.673 (0.544-0.802) | 0.75 |
| RV/LA ratio | 0.834 (0.722-0.946) | 0.804 (0.673-0.935) | 0.43 |

Values are given as area under the curve (95% confidence interval). Fully automatic cardiac chamber volumes were obtained without any manual correction. Axial diameters are identical to those reported in the main text and repeated here for comparison purposes.
